# Investigating the Role of RIO Protein Kinases in *Caenorhabditis elegans*

**DOI:** 10.1371/journal.pone.0117444

**Published:** 2015-02-17

**Authors:** Tasha K. Mendes, Stevan Novakovic, Greta Raymant, Sonja E. Bertram, Reza Esmaillie, Saravanapriah Nadarajan, Bert Breugelmans, Andreas Hofmann, Robin B. Gasser, Monica P. Colaiácovo, Peter R. Boag

**Affiliations:** 1 Department of Biochemistry and Molecular Biology, Monash University, Clayton, Victoria, Australia; 2 Institute for Genetics, University of Cologne, Cologne, Germany; 3 Department of Genetics, Harvard Medical School, Boston, Massachusetts, United States of America; 4 Faculty of Veterinary Science, The University of Melbourne, Parkville, Victoria, Australia; 5 Eskitis Institute for Cell & Drug Discovery, Griffith University, Brisbane, Australia; East Carolina University, UNITED STATES

## Abstract

RIO protein kinases (RIOKs) are a relatively conserved family of enzymes implicated in cell cycle control and ribosomal RNA processing. Despite their functional importance, they remain a poorly understood group of kinases in multicellular organisms. Here, we show that the *C. elegans* genome contains one member of each of the three RIOK sub-families and that each of the genes coding for them has a unique tissue expression pattern. Our analysis showed that the gene encoding RIOK-1 (*riok-1*) was broadly and strongly expressed. Interestingly, the intestinal expression of *riok-1* was dependent upon two putative binding sites for the oxidative and xenobiotic stress response transcription factor SKN-1. RNA interference (RNAi)-mediated knock down of *riok-1* resulted in germline defects, including defects in germ line stem cell proliferation, oocyte maturation and the production of endomitotic oocytes. Taken together, our findings indicate new functions for RIOK-1 in post mitotic tissues and in reproduction.

## Introduction

Protein kinases form a large family of diverse regulatory enzymes that are encoded by approximately two percent of the genes in most metazoan genomes [1, 2]. Through the phosphorylation of protein targets, they regulate various cellular processes, including transcription, translation and cell-cycle progression [1]. Of the 518 protein kinases encoded in the human genome, 478 form a single superfamily known as the eukaryotic protein kinase (ePK) family [1]. These enzymes are defined by their conserved, bi-lobed catalytic core which contains 12 subdomains involved in substrate binding, ATP binding and catalysis [3]. A second, smaller superfamily of 40 ‘atypical’ protein kinases (aPK) share structural homology to the ePK catalytic core, but lack overall sequence similarity [4]. The aPKs are divided into 13 small homology groups, one of which is the *ri*ght *o*pen reading frame (RIO) family of kinases.

Two members of the RIO kinase (RIOK) family, designated Rio1p and Rio2p, were identified in the yeast *Saccharomyces cerevisiae* [5, 6]. Subsequent studies have identified these kinases in various organisms, ranging from ancient single-celled archaea to complex multicellular eukaryotes [4, 7]. A third member named RIOK-3 that has greater similarly to RIOK-1 was first identified as a homolog of *Aspergillus nidulans* SUDD [8]. To date, RIOK-3 is only known to exist within multicellular eukaryotes [9]. The RIOK family feature a distinctive RIO domain that contains motifs typical of ePKs, including ATP-binding, catalytic and metal-binding loops and a hinge region [9, 10], but lacks motifs involved in substrate binding and the ‘activation’ domain. The truncated RIO catalytic domain and the inability to identify *in vitro* substrates for RIOK-1 and RIOK-2 have led to speculation that the RIOKs do not function as kinases *in vivo*, and are instead ATPases with vestigial kinase-related structures [11]. While RIOKs have been demonstrated to undergo autophosphorylation, their *in vivo* targets are unknown [9].

RIOKs have been reported to function in multiple pathways and links to various cancers and other human diseases are emerging [11–13]. RIOK-1 and RIOK-2 are non-ribosomal factors individually required for normal ribosomal RNA biogenesis and cell cycle progression [5, 14, 15]. In yeast, depletion of either RIOK-1 or RIOK-2 results in defects in 20S pre-ribosomal RNA processing. In human cells, RIOK-2 is required for the production of 18S pre-rRNA [15] and RIOK-3 is require for 21S pre-rRNA processing [16]. RIO-2 has also been identified to be a ribosomal assembly factor that prevents premature translation initiation on the small (40S) subunit [17, 18]. Depletion of yeast RIOK-1 results in a dramatic increase in the number of binucleated and anucleated cells and a disruption to G1 to S and anaphase progression [5]. In contrast, yeast cells depleted of RIOK-2 do not feature any stage specific cell cycle arrest; however, they show accelerated mitotic exit and a correlated increase in the degradation of the cell cycle regulator cyclin B1 [6]. Recently, RIOK-3 was shown to be an adapter protein required for NF-κB signaling [19] and for antiviral immune responses via the type I interferon pathway [20].

Although RIOKs have been studied in yeast and mammalian cell lines, presently little is known about them in a developmental and organismal context. Here, we report that the *C. elegans* genome contains three *riok* genes and that each of them has a distinct tissue expression pattern and that *riok-1* and-*2* are essential for development. We also show that *riok-1* is essential for reproduction, where it is required for oogenesis, but not spermatogenesis. Knockdown of *riok-1* by RNA interference (RNAi) results in the formation of endomitotic oocytes, suggesting a new role for RIOKs in meiosis.

## Materials and Methods

### Strains

*C. elegans* strains were cultured using standard techniques [21]. The wild-type strain Bristol N2 and the following mutant strains were used: *fog-2(q71)V*, *rrf-1(pk1417)I*, VP303 *rde-1*(ne219); kbIs7[pnhx-2::rde-1, rol-6]. The *riok-1(tm3775)* and *riok-2(tm3803)* deletion mutants where generated by the National BioResource Project for the Nematode, and were outcrossed at least seven times to N2 and maintained as balanced strains *riok-1(tm3775)/hT2* and *riok-2(tm3803)/hIn1*.

### RNAi

RNAi clones to *riok-1*, *dpy-13*, *car-1* and *skn-1* were obtained from the *C. elegans* ORFeome library [22]. We generated RNAi feeding constructs for *riok-2* and *riok-3* by cloning 1000 bp and 1200 bp amplicons produced by reverse transcription PCR (REF) into the feeding vector pL4440 and transformation into the RNAi feeding *bacterial strain* HT115(DE3). For feeding RNAi, synchronised L1s were placed on RNAi plates until they grew to adult hermaphrodites. As a negative control, we used the plasmid pCB19, which encodes a portion of the *Arabidopsis thaliana* gene Lhcb4.3 that has no homology to *C. elegans*.

### Analysis of brood size

Brood size analysis was completed at 20°C, 23°C and 25°C. Synchronised L1 worms were placed on RNAi plates and grown to L4 stage. At the L4 stage, were singled on to individual plates and transferred to a new small plate every 12 h. For the *fog-2* brood size, worms were grown on RNAi plates from the L1 stage and then one L4 female was transferred to a plate along with 10 males worms and the worms moved every 12 hours to a fresh plate

### Constructing transgenic worms

To create P_riok-1_2SKN-1_::GFP, a 2403 bp region 5’ of the ATG site and the first two exons of *riok-1* was amplified and cloned into the *Pst*I and *Bam*HI sites in pPD95.75 (total insert size: 2591 bp). A second construct containing one predicted SKN-1 site (P_riok-1_1SKN-1_::GFP), was created by amplifying a 1917 bp-region 5’ of the ATG site including the first two exons of *riok-1* followed by cloning into pPD95.75 (total insert size: 2142 bp). A third construct that lacked any SKN-1 site (P_riok-1_ ΔSKN-1_::GFP) was made following site-directed mutagenesis of P_riok-1_1SKN-1_::GFP using a QuikChange II site—directed mutagenesis kit (Stratagene). To achieve this, primers were designed to mutagenise the predicted SKN-1 site from att**G**t**CAT** to att**C**t**GCA**, which has been shown to inhibit SKN-1 binding [23]. To create P_riok-2_::GFP, a 703 bp region upstream of the ATG site and including the first two exons was amplified and cloned into the *Pst*I and *Bam*HI sites in pPD95.75 (total insert size: 2098 bp). To create P_riok-3_::GFP, 592 bp upstream to the ATG and the first four exon was amplified and cloned into the *Pst*I and *Bam*HI sites in pPD95.75 (total insert size: 2056 bp). All constructs were confirmed by sequencing. Wild-type worms were microinjected with the GFP reporter construct (50 ng/μl) and *rol-6* marker pRF4 construct (50 ng/μl) using standard methods [24].

### Imaging

For the immunostaining of gonads, worms were anesthetised in 0.001% tetramisole in M9 buffer. Gonads were dissected, snap frozen in liquid nitrogen and stained as described previously [25]. Slides were examined using an Olympus 1X81/1X2-UCB microscope. Primary antibodies used were: anti-PH3 (1/2000; rabbit) (Upstate Biotechnology), mAB414 (1/200; mouse) (Covance), MAPK (1/200; mouse) (Sigma). Secondary antibodies Alexa A488 and A555 (Invitrogen) were used at dilutions 1/1000 and 1/1200, respectively.

For live worm imaging, worms were anesthetized in a minimum of 0.001% tetramisole in M9 on a cover slip and mounted on a 1% agarose pad. Live worm slides were examined under the Olympus 1X81/1X2-UCB microscope or a Zeiss LSM 510 Meta confocal microscope. To observe sensory neurons in live RIOK::GFP transgenic worms, they were incubated with the lipophilic fluorescent dye Dil (Life Technologies) in the dark for three hours. Worms were then washed once with M9, plated and destained overnight and live worms were mounted on 1% agarose pads and examined by confocal microscopy. All images were processed using Adobe Photoshop and figures drawn using Adobe Illustrator.

## Results

### The *C. elegans* genome contains three *riok* genes

**Fig 1:**
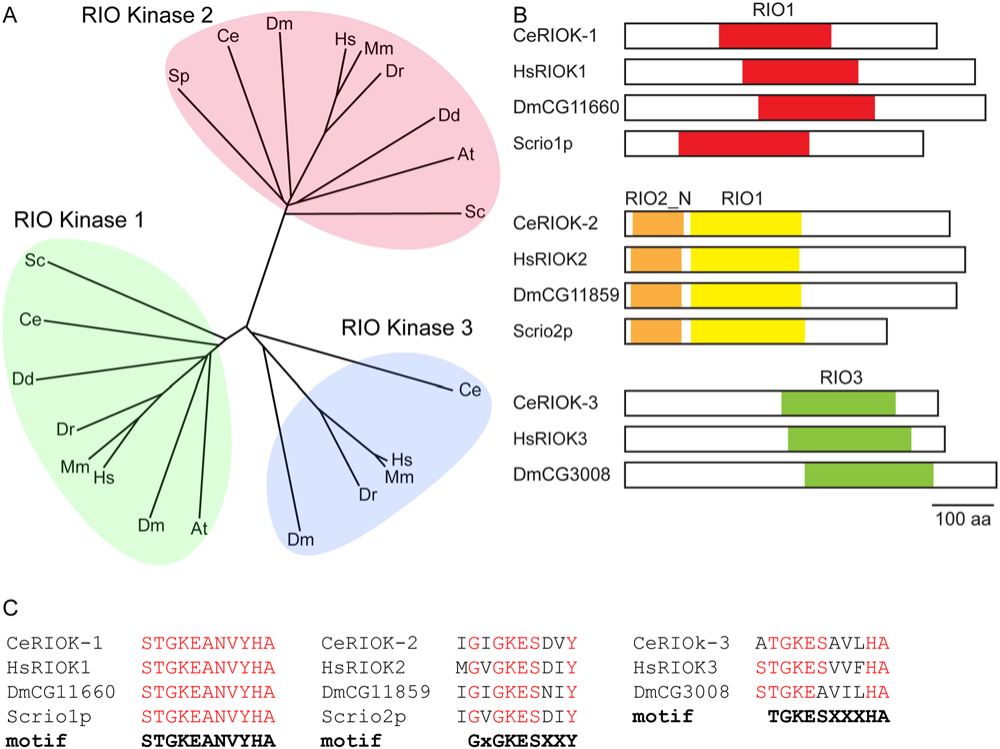
The RIO family of kinases are evolutionary conserved. (A) Unrooted phylogenetic tree of RIO proteins derived from a Clustal Omega alignment of whole proteins sequences (European Bioinformatics Institute). Tree was drawn using Philodendron Phylogenetic tree printer. Hs, *H. sapiens*; Mm, *M. musculus*; Dm, *melanogaster*; Ce, *C*. elegans; Xl, *X. Laevis*; Dr, *D. reri*; Dd, *D. discoideum*; At, *A. thaliana*; Sc, *S. cerevisiae* (B) Schematic representing the domain organisation in RIO kinases. Each of the RIO kinase sub-families has a conserved domain organisation; Hs, *H. sapiens*; Dm, *melanogaster*; Ce, *C*. elegans; Sc, *S. cerevisiae* examined. (C) Alignment of the ATP binding loop in the RIO domain.

The proteins M01B12.5/RIOK-1 and Y105E8B.3/RIOK-2 were encoded in the *C. elegans* genome, and have 61% and 56% similarity to the yeast homologs Rio1p and Rio2p, respectively. A third RIOK, protein ZK632.3/RIOK-3, was 48% similar to Rio1p. Phylogenetic analysis places *C. elegans* RIOK-1 and RIOK-2 with RIOK orthologs from yeast to humans, and RIOK-3 to a metazoan-specific group of RIOKs (Fig. 1A). Each of the three RIOKs has distinct domains (Fig. 1B) and regions of conservation (data not shown). RIOK-1 has a highly conserved “RIOK-like kinase” domain [10] in the middle of the protein. RIOK-2 kinases are most similar in the amino terminal half of the protein where two domains are identified: the RIOK-2 N-terminal domain which is similar to winged helix domains and may be able to bind DNA [10] and the RIOK-1 catalytic domain. RIOK-3 kinases are less conserved in sequence, but contain the “RIOK-like kinase” domain in the carboxy-terminal half of the protein.

The phosphate-binding loop (P-loop), which binds nucleotides, has been used to differentiate among the three RIOK families [26]. The P-loop of RIOK-1 kinase of *S. cerevisiae*, *Drosophila*, *C. elegans and humans are identical in sequence and match the* canonical RIOK-1 motif [26] (Fig. 1C). The P-*loop of* RIOK-2 *kinases are less well conserved, but adheres to the GxGKESxxY motif characteristic of RIOK-2 proteins. The RIOK-3 P-loop motif is the least well conserved, with both the C. elegans and Drosophila proteins having a serine to alanine change from the canonical motif for RIOK-3 P-loops (Fig. 1C). Taken together, these findings indicate that the C. elegans genome has a single member of each of the three RIOKs*.

### Localisation of *riok* expression

**Fig 2:**
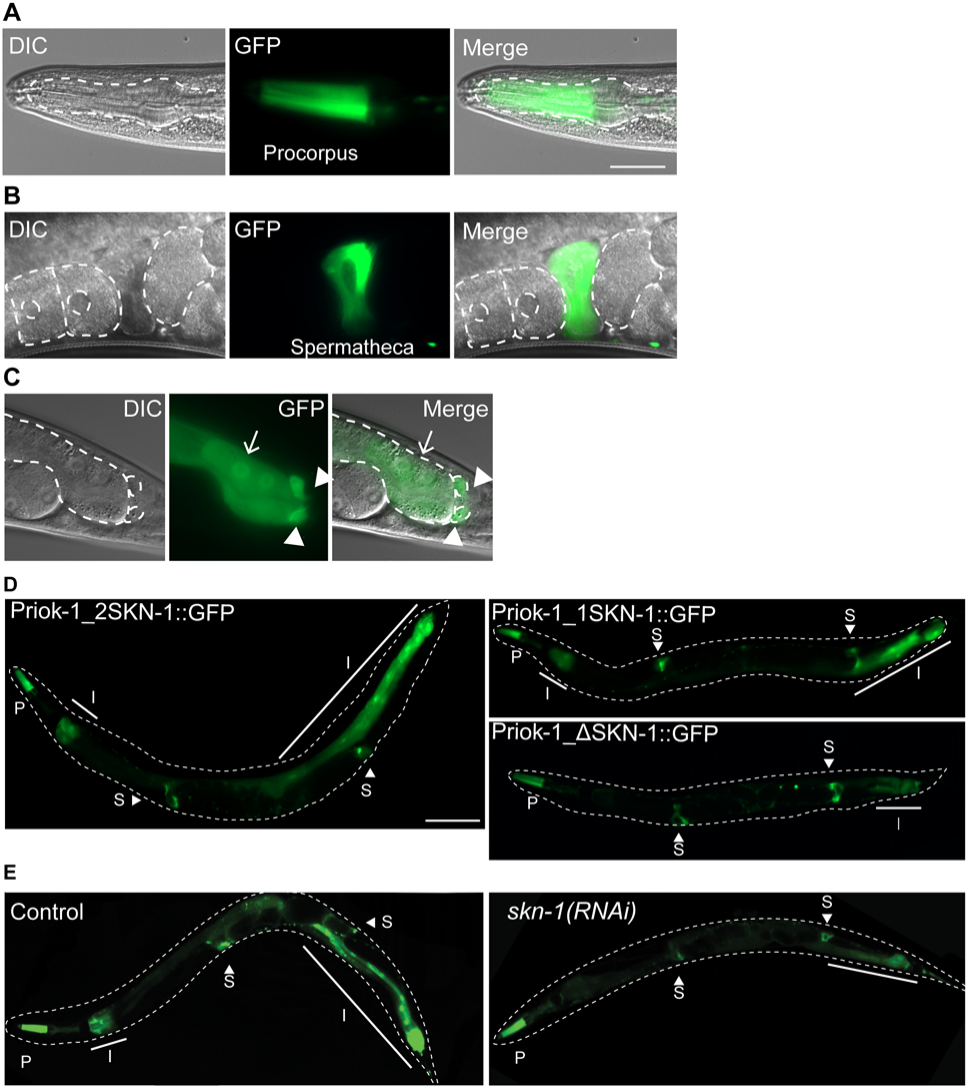
The putative *riok-1* promoter drives GFP expression in multiple tissues. (A) procorpus region of the pharynx; pharynx is outlined (B) spermatheca; oocytes and eggs are outlined. (C) intestine (arrow) and the rectal gland (arrow heads); intestinal cells and rectal gland outlined. Scale bar = 25μm. (D) Intestinal expression is largely SKN-1 dependent. Representative images of GFP expression in P*riok-1_2SKN-1*, P*riok-1_1SKN-1* and P*riok-1_ΔSKN-1* worms. I, intestine; P, Pharynx; S, spermatheca. (E) SKN-1 is required for normal expression of *riok-1* in the intestine. RNAi knockdown of *skn-1*, but not control (*pCB19)*, leads to a reduction in the P*riok-1_2SKN-1* intestinal expression. Worms are outlined with a dashed line and the solid line indicates the extent of GFP expression in the intestine. Scale bar = 100 μm.

The tissue expression pattern of the three members of the RIOK family is not known for any metazoans. Therefore, we generated transcriptional fusion reporters that express GFP under the control of each of the predicted *riok* promoter sequences. We defined the promoter of each gene as the intergenic region between the ATG site of the *rio*k gene and the nearest 5’ gene. A previous study [27] showed that knockdown of *riok-1* by RNAi induced expression of several target genes of the stress response transcription factor SKN-1. Given this connection we examined the putative *riok-1* promoter for SKN-1 binding sites. We identified two putative SKN-1 sites 512 (TTTATCAT) and 2312 nucleotides (ATTGTCAT) from the start of the *riok-1* open reading frame. The GFP expression pattern of both P_riok-1_2SKN-1_::GFP and P_riok-1_::GFP were similar in all developmental stages. Expression was detected in the pharynx (procorpus), the spermatheca, intestine, some neurons, rectal gland and the rectal valve (Fig. 2 and data not shown). In the intestine, GFP expression by the promoter containing two SKN-1 binding sites (P_riok-1_2SKN-1_::GFP) was slightly more extensive compared to the P_riok-1_1SKN-1_ promoter (Fig. 2D). We generated additional transgenic lines in which the predicted SKN-1 binding site from the P_riok-1_1SKN-1_::GFP construct was changed from ATT**G**T**CAT** to ATT**C**T**GCA**, as these mutations have been shown to abolish SKN-1 binding [23]. In these transgenic lines GFP expression was unchanged in non-intestinal cells, while intestinal cells showed dramatically reduced GFP expression (Fig. 2D). To further examine the requirement for SKN-1 for *riok-1* gene expression, we used RNAi to knockdown SKN-1 levels. In *skn-1(RNAi)* worms, the intestinal expression of the P_riok-1_2SKN-1_::GFP reporter gene was significantly reduced in the intestine cells, while expression in the other somatic tissues was unchanged (Fig. 2E). We conclude that high levels of GFP expression in the intestine, but not other tissues, requires SKN-1 and SKN-1-binding sites in the *riok-1* promoter.

**Fig 3:**
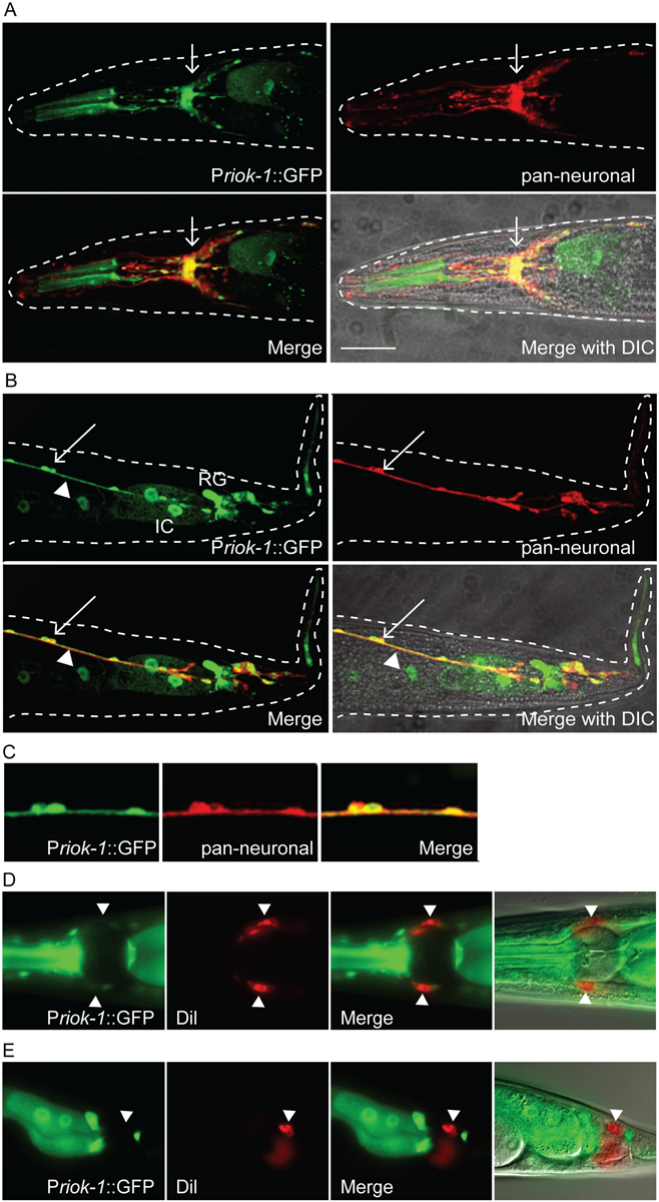
P*riok-1_2SKN-1* drives GFP expression in a small subset of neurons. (A–C) Single plane confocal images from adult worms. (A) GFP was expressed in a subset of head neurons and is highly expressed in the nerve ring displaying strong co-localisation with the pan-neuronal marker (arrow). (B) Co-localisation of GFP with pan-neuronal dsRED. D-type neurons (arrow) and longitudinal nerves (arrow head). Intestinal cells (IC) and the rectal gland (RG) can also be observed. (C) Zoomed in image of the D-type neurons displaying co-localisation. Scale bar = 25μm. (D–E) P*riok-1_2SKN-1* GFP expression does not co-localise with the lipophilic dye Dil, in head neurons (D) or tail neurons (E). Scale bar = 15 μm.

To confirm the neuronal expression pattern of P_riok-1_::GFP, we generated transgenic worms expressing both P_riok-1_::GFP and a pan-neuronal marker (P_*rgef-*_ *1*::*DsRed2*). Co-localisation of GFP with the pan-neuronal marker was observed in tail and longitudinal neurons and in all D-type neurons (Fig. 3). P_riok-1_1SKN-1_::GFP reporter gene expression was observed in a subset of neurons within the head and tail regions of worms. To determine whether these neurons are sensory, we conducted dye-filling assays. No co-localisation of the lipophilic dye (Dil) with GFP was detected (Fig. 3 D-E), indicating that RIOK-1 is not expressed in the head (amphid) or the tail (phasmid) sensory neurons in *C. elegans*.

**Fig 4:**
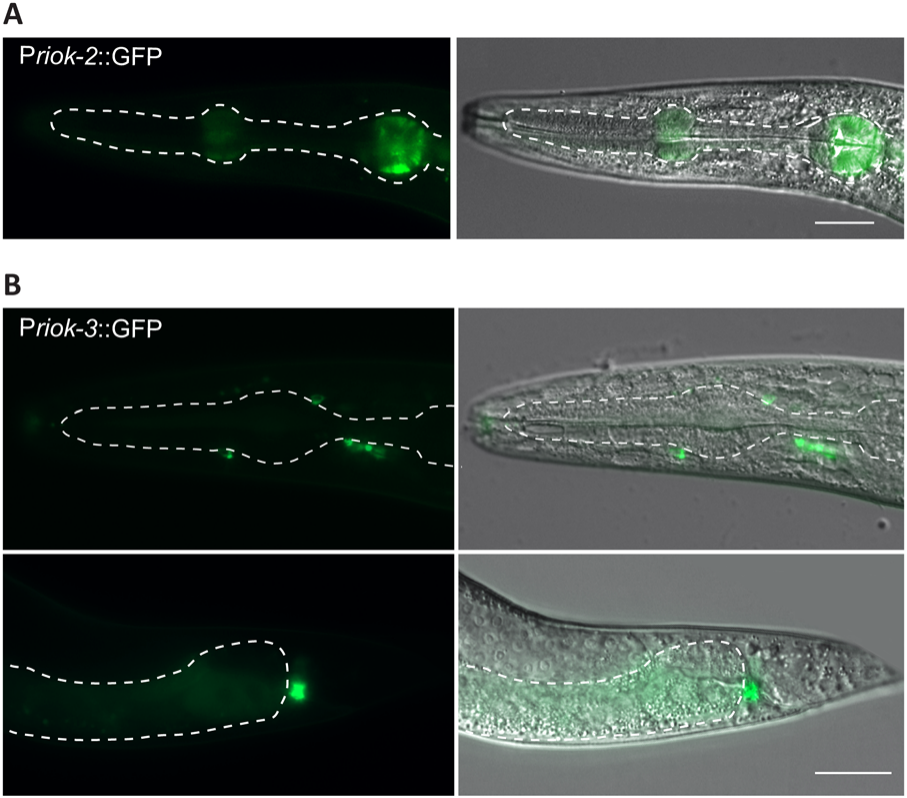
The putative promoters of *riok-2* and -*3* drive GFP in distinct tissues. (**A)** *riok-2* transgenic worms displayed weak GFP expression in the metacorpus and posterior bulb of the pharynx. Scale bar = 25μm (B) *riok-3* transgenic worms revealed weak GFP expression, in some head and was strongly expressed in a tail neuron which may be the PVQ, PHAL or PQR neuron. Scale bar = 25μm.

In contrast to RIOK-1, both RIOK-2 and-3 have a restricted tissue expression pattern. Worms expressing GFP under the control of the *riok-2* promoter showed consistent GFP expression only in adult staged worms, where it localised to the metacorpus and posterior bulbus of the pharynx (Fig. 4A). GFP expression under control of the *riok-3* promoter was first observed in embryos and continued throughout larval development. GFP intensity was highest in adult worms, where it was found in a small number of tail neurons whose positioning was consistent with PVQ, PHAL/PQR neurons (Fig. 4B).

### Functional analysis of RIOKs

**Fig 5:**
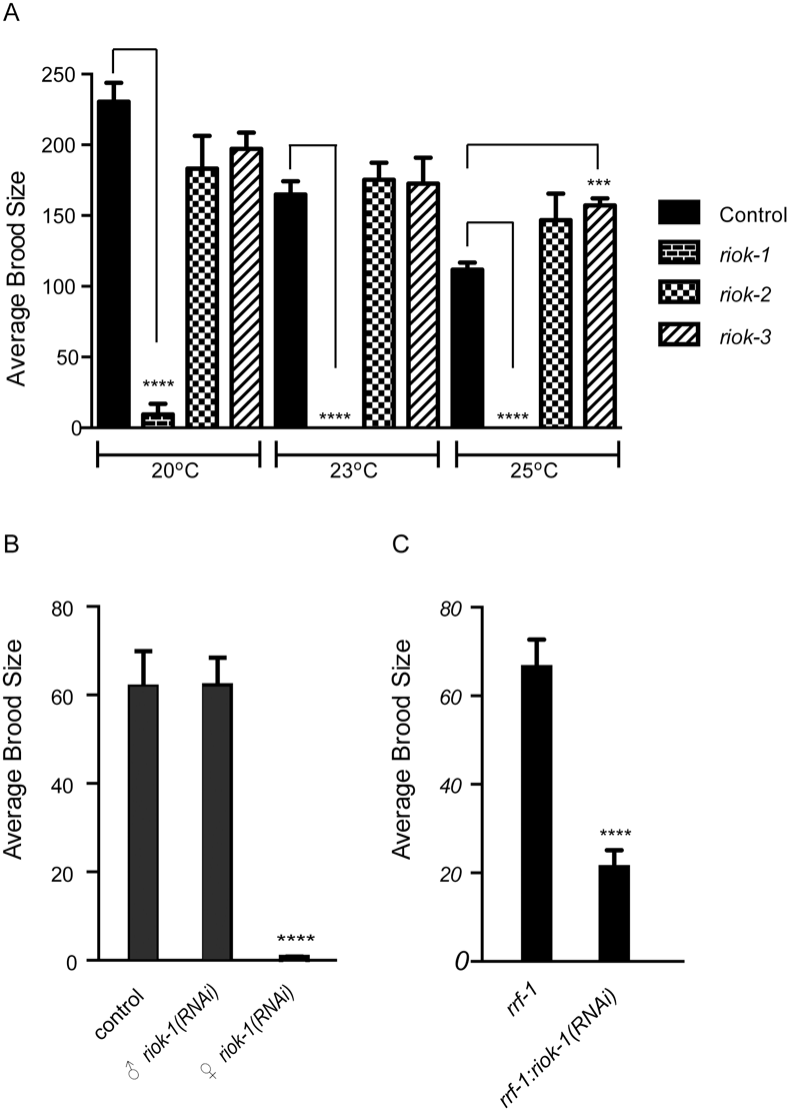
*riok-1(RNAi)* sterility is associated with an oogenesis defect. (A) Total average brood size for wild-type worms knocked down for r*iok-1*, *riok-2* or *riok-3* at 20°C, 23°C and 25°C. *riok-1(RNAi)* worms have a significant reduction in brood size at 20°C and are sterile at 23°C and 25°C. Error bars represent the SEM; *n* ≥*6*. _****_ = P values <0.0001. _***_ = P values 0.0003 for *riok-3* at 25°C (B) Average number of progeny produced in 17 hours from crossing *fog-2(q71)* male and female worms. Crosses were; control, ♀ *fog-2(q71)* x *♂ fog-2(q71)*; ♀ *fog-2(q71)* x *♂riok-1(RNAi)*; ♀ *fog-2(q71)riok-1(RNAi)* x *♂ fog-2(q71)*. Thirteen individual crosses per treatment were conducted at 25°C with 10 males mated per L4 female. _****_ = P <0.0001. (C) Knockdown of *riok-1* in *rrf-1* worms resulted in significantly reduced progeny. Total brood size for *rrf-1* worms grown on *riok-1* and pCB19 (negative control) RNAi at 25°C. Error bars represent SEM. *n = 23*, _****_ = P <0.0001.

The functions of RIOKs in *C. elegans* are not yet understood. Predicted genetic null mutants are available for all three *rioks*; homozygote mutants for *riok-1* and *riok-2* both had 100% penetrance early larval arrest, while *riok-3* worms had no obvious phenotype (data not shown). To characterise the functions of *riok-1*, *riok-2* and *riok-3*, we used RNAi to knockdown each kinase. RNAi by microinjection led to early larval arrest similar to the null mutants representing *riok-1* and *riok-2*, but no overt phenotypes for *riok*-3. RNAi using the feeding approach produced worms that developed to adulthood without any obvious developmental deficits. However, knockdown of *riok-1* resulted in a significant reduction in the number of progeny produced at 20°C and sterility at 23°C and 25°C. Knockdown of *riok-2* and *riok-3* produced similar numbers of progeny compared with their controls (Fig. 5A). Given the neuronal expression of *riok*-1, we also conducted behavioral touch assays using neuronal RNAi sensitive strains. However, we did not observe any touch response defects (data not shown), suggesting that *riok*-1 is not essential for this response. The relatively broad tissue expression pattern of *riok-1* in adult worms and its requirement for reproduction indicate that this kinase may have diverse functions in *C. elegans*.

To establish whether sterility linked to RIOK-1 was due to a defect in spermatogenesis or oogenesis, we knocked down *riok-1* in *fog-2(q70)*, which exists as a male and female worms. When *fog-2* female worms were knocked down for *riok-1* and crossed with untreated male worms, very few (10 ± 2, *n* = 10) progeny were generated. In contrast, *riok-1(RNAi)* male worms crossed with untreated females produced high levels of progeny (Fig. 5B). Based on these findings, we conclude that *riok*-1 is essential for normal oogenesis but dispensable for spermatogenesis. To determine whether the *riok-1(RNAi)* oogenesis defect was somatic gonad or germ cell-derived, we took advantage of a mutant in *rrf-1* which is resistant to RNAi in most somatic tissues, but capable of RNAi in germ cells. Knockdown of *riok-1* in *rrf-1* worms resulted in 48% of worms being sterile and 52% producing few progeny (21.7 ± 3.3 *n* = 34, P<0.01) (Fig. 5C). The *rrf*-1 strain does have some intestinal RNAi capacity [28], therefore we knocked down *riok-1* in the stain VP303, which is only capable of RNAi in intestinal cells. When *riok-1* was knocked down in this background the worms had a normal brood size (data not shown). We concluded that the *riok-1* sterility likely results from a defect in germ cell function during oogenesis.

### RIOK-1 is required for normal germ cell proliferation

**Fig 6:**
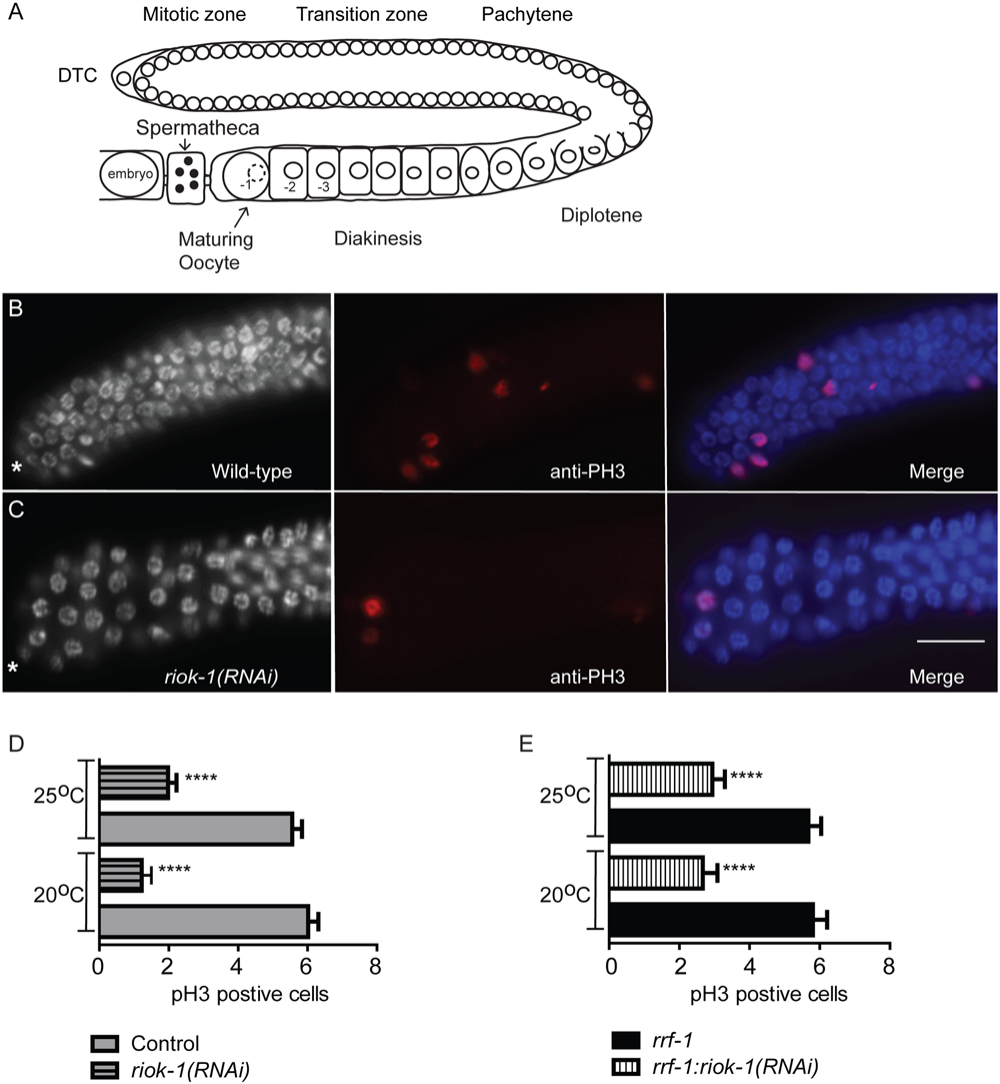
*riok-1* is required for normal germ cell proliferation. (A) Overview of the adult *C. elegans* hermaphrodite gonad. One of the two gonad U-shaped tubular gonad is shown. The single somatic distal tip cell (DTC) maintains a population of self-renewing germline stem cells. Germ cells enter into the meiotic prophase I in the transition zone and then progress to the pachytene stage. During these stages, germ cells are only partially enclosed in a membrane and share a common cytoplasmic core. As germ cells exit pachytene, they move through the “loop” region and enter diplotene and begin to fully cellularise. A signal from sperm directs the most proximal oocytes to undergo maturation and fertilisation occurs are the oocyte moves through the spermatheca into the uterus. (B-C) Dissected gonads were stained with anti-pH3 staining to detect proliferating germ cells and DAPI to visualise DNA. Asterisks indicate the distal end of the gonad. Scale bar = 15μm. (D-E) *riok-1(RNAi)* gonads had a reduced number of pH3 stained cells in the mitotic zone (D) Wild-type worms with *riok-1* knocked down at 20°C and 25°C *(n = 50)* (E)) *rrf-1;pCB19(RNAi)* and *rrf-1*:*riok-1(RNAi)* worms at 20°C and 25°C *(n = 50)*. Error bars represent SEM; _****_ = P values <0.0001.

We next examined the gonads of one-day old hermaphrodites depleted of RIOK-1, -2 or-3. Gonads of *riok-2(RNAi)* and *riok-3(RNAi)* worms displayed grossly normal morphology, whereas *riok-1(RNAi)* worms exhibited smaller mitotic and transitions zones in the distal gonad compared with controls (Table 1). We then examined whether knockdown of *riok-1* caused a reduction in cellular proliferation by immunostaining dissected gonads with the proliferation marker anti-phosphohistone H3 (PH3). Worms knocked down for *riok*-1 had a significantly reduced number of PH3-positive cells in the mitotic zone at both 20°C and 25°C (Fig. 6B-D), indicating decreased germ cell proliferation. To examine whether this decrease was due to *riok-1* expression in somatic or germ cells, we knocked down *riok*-*1* in a strain lacking the *rrf-1* gene. Germ cell proliferation was indeed reduced in *rrf-1*:*riok-1(RNAi)* compared with *rrf-1* worms (Fig. 6E), suggesting that the observed gonadal defects are likely to be due to germ rather than somatic cell abnormalities. Interestingly, no defects in chromosomal organisation were detected when gonads of *riok-1(RNAi)* worms were examined for defects in transition zone and pachytene stage germ cells using antibodies to the synaptonemal complex proteins SYP-1 [29](S1 Fig.), suggesting that *riok-1* is not required for the pachytene stage of germ cell development. Together, these data indicate that *riok-1* is required for the early stages of germ cell development.

**Table 1:** Worms depleted of *riok-1* had a significant reduction in the size of the mitotic zone.

	Distal germ cells count (diameter)	
	Mitotic zone (MZ)	Transition zone (TZ)
**Wild-type**	19 cells ± 0.4 *(n = 10)*	9 cells ± 0.3 *(n = 10)*
***riok-1(RNAi)***	12 cells ± 0.7 *(n = 10)*	7 cells ± 0.4 *(n = 10)*

Values represent Mean and SEM (P <0.0001).

### RIOK-1 is required for normal germ cell progression in the proximal gonad

**Fig 7:**
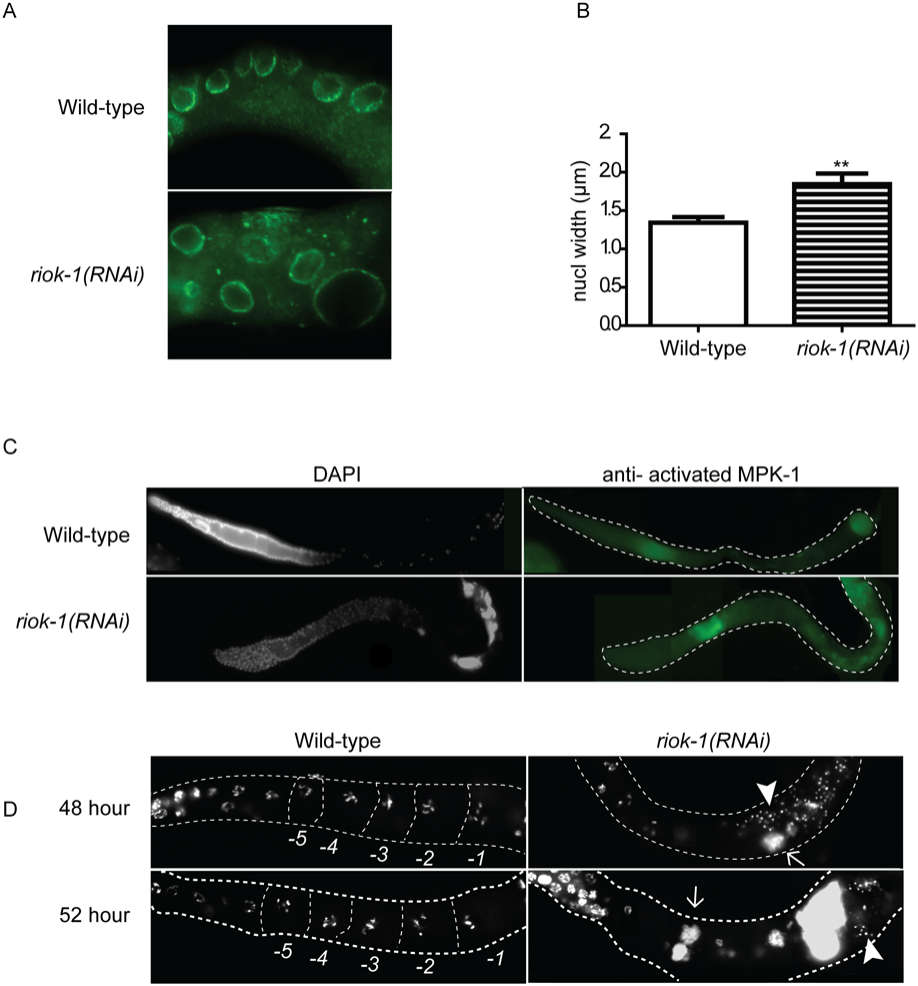
Proximal gonad organization requires *riok-1*. (A) Representative images of wild-type and *riok-1(RNAi)* dissected gonads immunostained stained for nuclear pores. (B) The nuclear diameter of germ cells in the proximal loop region of *riok-1(RNAi)* worms are significantly increased in size *(n = 12*). Error bars represent SEM; _**_ = P values 0.0045. (C) Wild-type and *riok-1(RNAi)* dissected gonads immunostained stained for activated MAPK. Increased MAPK staining is seen in pachytene and throughout diplotene. (D) Progression of the *riok-1(RNAi)* EMO phenotype was examined at time points 48 and 52 hours post L1. Gonads from dissected wild-type and *riok-1(RNAi)* worms were stained with DAPI to visualise DNA. The gonad and oocytes are outlined and proximal wild-type oocytes have been outlined and labelled (-1 to-5). At all time points wild-type worms displayed the typical six bivalent chromosomes in their maturing oocytes within the proximal gonad *(n = 30). riok-1(RNAi)* worms *(n = 30)* showed DNA aggregates (arrow) in the proximal region of the gonad and abnormal localised sperm (arrow head). Scale bar = 25 μm; worms grown at 25°C.

After germ cells exit pachytene, they enter the loop region of the gonad where they progress into the diplotene stage. In wild-type worms, germ cells are organised into a single row as they move through diplotene (Fig. 6A). When *riok-1* was knocked down, germ cells entering diplotene were disorganised and their nuclei were abnormally enlarged. We immunostained dissected gonads with a nuclear pore complex marker and found that germ cell nuclei of *riok-1(RNAi)* worms were significantly larger (P<0.05) than wild-type worms (Fig. 7A-B). As germ cells exit pachytene, MPK-1 mitogen activated protein kinase (MAPK) is inactivated and only reactivated in the most proximal oocytes. To examine whether this down regulation of MAPK activity occurs in the absence of *riok-1*, we immunostained gonads from one-day old *riok-1(RNAi)* worms with a monoclonal antibody that specifically binds to the activated, diphosphorylated form of MAPK (dpMPK-1). In wild-type worms, MAPK staining was predominantly present in the pachytene germ cells and the most proximal oocytes (Fig. 7C). Interestingly, in 50% (*n* = 120) of *riok-1(RNAi)* gonads, dpMPK-1 staining did not decrease as germ cell exit pachytene, instead it was evident throughout diplotene and diakinesis (Fig. 7C). These findings indicate that RIOK-1 is directly or indirectly required to inhibit the MAPK pathway as germ cells exit pachytene or limit the activation in late stage oocytes.

Oocytes in *riok-1(RNAi)* worms appeared abnormal and did not display the typical rectangular shape seen in wild-type worms. Large DNA aggregates were detected in the proximal gonad of *riok-1* knockdown worms (Fig. 7D). These DNA clumps were consistent with an endomitotic oocyte (EMO) phenotype [30]. This finding suggests that *riok-1* might be involved in regulating normal oocyte maturation or possibly ovulation. To examine the onset and progression of the endomitotic oocytes, gonads were dissected from wild-type and *riok-1(RNAi)* adult worms (48, 52, 56 and 60 h following the L1 stage). Wild-type worms displayed the typical six bivalent chromosomes within their oocytes at all time points (Fig. 7D). At 48 h, *riok-1(RNAi)* worms displayed small DNA aggregates (Fig. 7D, arrow) and they continued to get larger over time. At all time-points, *riok-1(RNAi)* worms had abnormally located sperm near the DNA clumps in the proximal gonad (Fig. 7D, data not shown). This result suggested that the spermatheca did not function normally when *riok-1* was perturbed. Temporal analysis showed that the *riok-1(RNAi)* DNA aggregates enlarged over time, indicating the EMO phenotype is aggravated.

To examine whether the EMO phenotype was sperm dependent, *riok-1* was knocked down in the male and female *fog-2(q70)* strain. In the hermaphrodite gonad spermatogenesis occurs prior to oogenesis, and oocyte maturation and ovulation occurs constitutively in adults until sperm stores are depleted [31]. In unmated female worms oocytes arrest in meiotic prophase I. No endomitotic oocytes were observed in *fog-2* (mated or unmated) control worms (Table 2). Within unmated *fog-2*: *riok-1(RNAi)* females, most worms did not display an EMO phenotype (Table 2). Surprisingly, mated *fog-2* females also did not display an EMO phenotype but were sterile (Table 2). Together these data suggest that the EMO phenotype is dependent upon hermaphrodite derived sperm, but not male derived sperm.

**Table 2:** Ovulation is required for endomitotic oocytes in *riok-1* worms.

Genotype	% EMO	*n-value*
Wild-type	0%	25
riok-1(RNAi)	100%	25
*fog-2(q71)* female unmated	0%	30
*fog-2(q71):riok-1(RNAi)* female unmated	0%	25
*fog-2(q71)* female mated	0%	27
*fog-2(q71):riok-1(RNAi)* female mated	0.1%	28

## Discussion

In this study, we showed that the *C. elegans* genome encodes three members of the RIOK family, and that RIOK-1 and-2 are essential for larval development. RIOKs from yeast and mammalian cells have been shown to be required for fundamental steps in rRNA maturation and cell cycle regulation [26]. Therefore, we were surprised that our transgenic analysis of *riok* gene expression revealed detectable expression in relatively few cells. It is possible that riok gene(s) are expressed in the germline, however our transgenic analysis would have not supported germline expression due to the silencing of the extrachromosomal transgene arrays in the germline [32]. Both *riok-1* and -*3* were expressed throughout development and adulthood, while *riok-2* was only detected in adult-stage worms. It is possible that the tissue expression pattern of the RIOKs is wider than our analysis showed when the expression specific levels are very low. Indeed, both Rio1P and Rio2P are reported to be expressed at very low levels in yeast [6, 33]. Therefore, it is possible that *riok-1* and-*2* are expressed widely, but at very low levels in *C. elegans* and may function in rRNA processing, as is the case in other species. Null mutants in other genes that affect rRNA processing in *C. elegans* also show a similar larval arrest as *riok-1* and-*2* mutants [34].

The *riok-1* promoter drove strong GFP expression in several neurons, the spermatheca and the intestine. The intestine is the primary detoxification organ in *C. elegans*. Expression of the *riok-1* reporter was high in the first anterior and the last few posterior cells of the intestine, a pattern similar to several genes that function in the oxidative stress pathway [27]. The *riok-1* intestinal expression was dependent on the predicted SKN-1 binding sites in its promoter. SKN-1 is a key transcription factor that is involved in oxidative and xenobiotic stress responses in *C. elegans* [35, 36]. Knockdown of *riok-1* has been reported to result in the induction of several genes in the SKN-1 stress response pathway, suggesting that *riok*-1 may function in regulating cellular metabolism, although *riok-1(RNAi)* worms showed only a modest decrease in stress resistance [27]. The genomic locus for *riok-1* encodes two isoforms of RIOK-1 [37]. The small isoform lacks the complete RIO domain, including the putative catalytic site. It will be interesting to develop tools to examine which of the two isoforms of RIOK-1 are expressed in a SKN-1-dependent manner, as the short isoform could act as an endogenous dominant-negative as has been reported for other protein kinases [38]. Together, these data suggest a new role for RIOKs in stress pathways.

An unexpected discovery was that *riok-1* is required for fertility. Worms in which *riok*-1 was knocked down displayed an oogenesis-specific sterility that was associated with several defects in gonad organisation. When *riok-1* was knocked down, the number of proliferating mitotic cells was significantly reduced, and the nuclei of germ cells entering the diplotene stage of meiosis prophase I had abnormally organised and large nuclei. In the proximal gonad, oocyte maturation appeared abnormal, and oocytes underwent multiple rounds of DNA replication, resulting in the generation of endomitotic oocytes. Interestingly, pachytene stage germ cells exhibited no obvious abnormalities, suggesting *riok-1* is required for several specific steps in germ cell development. As endomitotic oocytes are often associated with defects in oocyte ovulation and cell cycle regulation, some of the *riok-1* associated defects could be due to a direct role in cell cycle regulation as has been reported in mitotic cells of other organisms [5, 14, 15].

Normally, oocytes and sperm are kept physically separated, and they meet only as the oocytes enter the spermatheca during ovulation. The presence of sperm in the proximal gonad of *riok-1(RNAi)* worms might have contributed to the endomitotic phenotype through an inappropriate activation of the MAPK signaling, which was abundant throughout the proximal gonad (Fig. 7B). The *riok-1* reporter was highly expressed in the spermatheca but not in other tissues of the somatic gonad. RIOK-1 may play an important role in regulating the ability of the spermatheca to retain sperm, and thereby indirectly restrict MAPK signaling. The analysis of *riok-1* knockdown in the somatic RNAi deficient strain *rrf-1* led to 48% of worms being sterile, with the remainder producing very few progeny. Given that *riok-1* knockdown in wild-type worms consistently produced 100% sterility, it is possible that RIOK-1 does function in both somatic and germ cells, with the latter having a more dominant contribution to the sterility. Determining the gonad expression pattern of RIOK-1 might help resolve the relative contribution of these tissues. Taken together, our findings suggest that RIOKs in *C. elegans* have functional roles in both somatic and germ cells.

## Supporting Information

S1 Fig The kinetics of SC assembly are not affected in *riok-1(RNAi)* meiotic nuclei. High magnification images of wild-type and *riok-1(RNAi*) germline nuclei at the indicated stages stained with DAPI and SYP-1. Bars, 5 μm.
(TIF)
