# Correction: Investigating the Role of RIO Protein Kinases in *Caenorhabditis elegans*

**DOI:** 10.1371/journal.pone.0156191

**Published:** 2016-05-18

**Authors:** Tasha K. Mendes, Stevan Novakovic, Greta Raymant, Sonja E. Bertram, Reza Esmaillie, Saravanapriah Nadarajan, Bert Breugelmans, Andreas Hofmann, Robin B. Gasser, Monica P. Colaiácovo, Peter R. Boag

The following information is missing from the Funding section: This work was supported by a fellowship from the Lalor Foundation (S Nadarajan), National Institutes of Health grant R01GM072551 (MPC) and a National Health and Medical Research Council grant APP1044022 (RBG, PRB & AH).
