# Supplementary figures and images for: Investigating the Role of RIO Protein Kinases in Caenorhabditis elegans
Source: PLoS One. 2015 Feb 17;10(2):e0117444. doi: 10.1371/journal.pone.0117444 (PMC4331490; doi:10.1371/journal.pone.0117444)

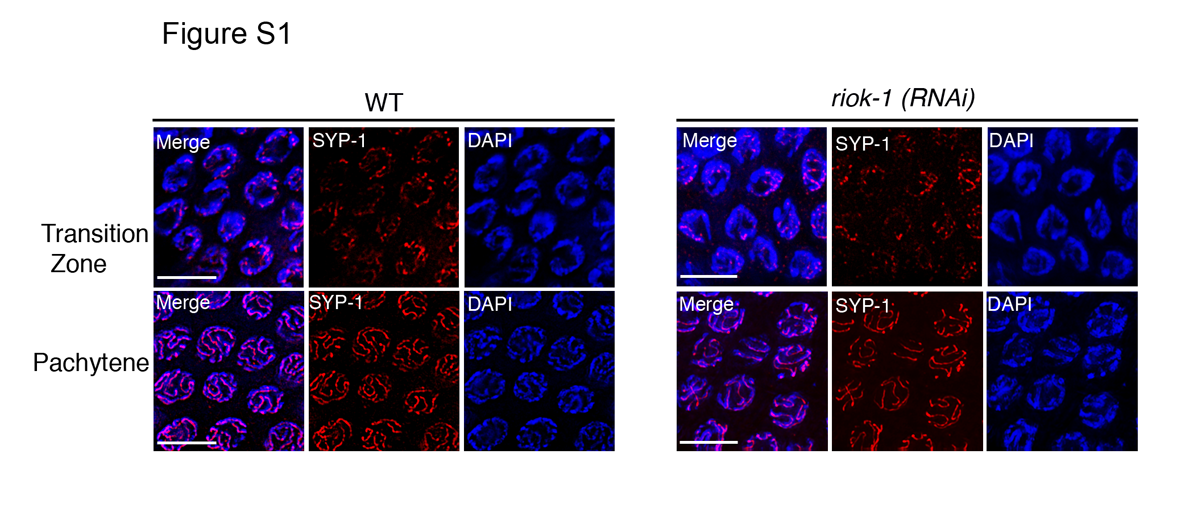

Supplement: S1 Fig — High magnification images of wild-type and riok-1(RNAi) germline nuclei at the indicated stages stained with DAPI and SYP-1. Bars, 5 μm. (TIF) [file pone.0117444.s001.tif]
